# Neoadjuvant Therapy in Nonmetastatic Breast Cancer in Kurdistan, Iraq

**DOI:** 10.1200/GO.22.00276

**Published:** 2023-05-22

**Authors:** Karez Sarbast Namiq, Luqman Rahman Sulaiman

**Affiliations:** ^1^ Department of Medicine, College of Medicine, Nanakali Hospital for Blood Diseases and Cancer, Hawler Medical University, Erbil, Iraq

## Abstract

**PURPOSE:**

The core management of nonmetastatic breast cancer includes surgical tumor removal by either breast-conserving surgery (BCS) or mastectomy. The use of neoadjuvant chemotherapy (NACT) has shown the potential to downstage locally advanced breast cancer (LABC) and reduce the extent of breast or axillary surgery. This study aimed to assess the treatment approach for nonmetastatic breast cancer in the Kurdistan region of Iraq and to compare its alignment with the current international recommendations for cancer treatment.

**METHODS:**

We retrospectively reviewed the records of 1,000 patients with prespecified eligible inclusion criteria who underwent either BCS or mastectomy for nonmetastatic invasive breast cancer at oncology centers in the Kurdistan region of Iraq between the period 2016 and 2021.

**RESULTS:**

Of 1,000 patients (median age, 47 years [range, 22-85 years]), 60.2% underwent mastectomy and 39.8% underwent BCS. The proportion of patients treated with NACT has increased over time, with 8.3% of patients receiving neoadjuvant treatment in 2016 compared with 14.2% in 2021. Similarly, BCS increased from 36.3% in 2016 to 43.7% in 2021. Most patients who underwent BCS had early breast cancer with low nodal involvement burden.

**CONCLUSION:**

The increasing trends of BCS practice in LABC along with the increased use of NACT in the Kurdistan region in recent years comply with international guidelines. Our large multicenter, real-life series emphasizes the need to implement and discuss more conservative surgical approaches, enhanced with the broader use of NACT, through education and information programs for health providers and patients, in the context of multidisciplinary team discussions, to deliver high-quality, patient-centric breast cancer care.

## INTRODUCTION

Breast cancer is one of the most common cancers worldwide and the most commonly diagnosed malignancy in women, internationally.^1^ The International Agency for Research on Cancer states that breast cancer represents one in four cancer types diagnosed in women, with an estimated 2,261,419 (24.5%) new breast cancer cases diagnosed in 2020.^2^ Cancer is the main cause of death in Eastern Mediterranean Region countries, including Iraq.^3^ In 2020, breast cancer accounted for 22.2% of all recently detected cancers, 37.9% of female malignancies, and 15.3% of cancer-related deaths among Iraqi female patients,^4^ similar in incidence to Syria, the United Arab Emirates, and Jordan.^2^

**CONTEXT**

**Key Objective**
To examine clinical trends in breast cancer care in the Kurdistan Region of Iraq.
To review the local stage distribution and treatment methods for patients with nonmetastatic breast cancer.
To identify shifts in the therapeutic paradigm and assess adherence to international guidelines.
**Knowledge Generated**
This study, comprising a large series of 1,000 patients, provides a comprehensive assessment of breast cancer care in the Kurdistan Region of Iraq. The study reveals a gradual but significant shift in the therapeutic paradigm toward conservative breast surgery due to increased usage of neoadjuvant therapy. However, the majority of patients continue to undergo radical surgery, even when they could be candidates for breast-conserving surgery. The study also highlights the lack of adherence to international guidelines with respect to neoadjuvant therapy and the absence of multidisciplinary discussion in treatment decision making.
**Relevance**
This study sheds light on the current state of breast cancer care in the Kurdistan Region of Iraq and provides important insights for healthcare professionals and policymakers in the region. The findings underscore the need for greater adherence to international guidelines, including the implementation of neoadjuvant therapy and multidisciplinary discussion in treatment decision making. The study highlights the potential for conservative breast surgery to be more widely adopted, leading to improved patient outcomes and quality of life. Finally, this study highlights the need for continued research in this area to further optimize breast cancer care in the Kurdistan Region of Iraq.

Locally advanced breast cancer (LABC) is a heterogeneous group of breast tumors with a locoregional spread that may be operable (stages IIB and IIIA) or potentially inoperable (stages IIIB and IIIC) without any clinicoradiologic evidence of metastasis.^5^ The lack of breast health care education in developing countries leads to the advanced stages of breast cancer. In Iraq and our region, a majority of population is diagnosed at a later stage.^6-8^

Breast cancer management involves a multidisciplinary approach. Pretherapeutic staging is based on a multitude of triple assessments of palpable breast lumps, including clinical examinations, imaging, and laboratory techniques.^9,10^ Histologic diagnosis and pathologic evaluation of essential markers, such as hormone receptors (HRs) and human epidermal growth factor receptor 2 (HER2), are critical for breast cancer treatment.^9,11^

For women with LABC, multimodal treatments aim to optimize locoregional disease control and eradicate occult systemic metastases. Neoadjuvant therapy was first introduced in the 1970s.^12^ Neoadjuvant chemotherapy (NACT) is defined as the administration of chemotherapy before definitive surgery, which is now widely used for patients with early- and locally advanced-stage breast cancer.^13^ NACT is usually followed by locoregional management and has been successfully used in clinical practice to minimize the extent of breast surgery, downstaging, and shrinking tumor size.^11,14,15^ Another benefit of NACT is the opportunity to de-escalate axillary nodal surgery.^16^

Neoadjuvant treatment is becoming the standard approach in many institutes and is usually offered to younger patients, clinically large tumor sizes, and node-positive, multifocal, and multicentric breast cancer. In addition, patients with triple-negative or HER2-positive breast cancer are usually recommended to be treated with NACT.^17^

For eight decades, radical mastectomy has been the only option for breast cancer surgery.^18^ In 1969, the term quadrantectomy was approved by the World Health Organization.^19^ Subsequently, breast-conserving surgery (BCS) plus adjuvant radiation therapy has been proven to be equivalent to mastectomy.^20^ Since then, the standard surgical procedures for breast cancer management have included either BCS or mastectomy with axillary dissection.^21^

The LABC management is complex^22^ and requires all appropriate specialties in a multidisciplinary team (MDT), including radiologists; pathologists; surgical, medical, and radiation oncologists; gynecologists; oncology psychologists; social workers; nursing teams; nutritionists; and palliative care specialists.^23^ Once a patient has been identified for NACT, a multimodal radiologic assessment before and during NACT is essential to assess tumor response.^23^ Pretherapy radiologic marker clips can be placed in the breast and any biopsy-proven positive nodes to help the surgeon to locate the tumor during the surgery and have less extensive axillary lymph node dissection (ALND).^24^

Unlike most cancer treatment programs worldwide that prioritize BCS in the early cancer stages, mastectomy is widely embraced over BCS in our region, regardless of the breast cancer stage at the time of diagnosis.^25^ Globally, studies have provided an elaborate comparison of BCS and mastectomy to encourage patients with breast cancer to consider BCS in breast cancer management as an equally beneficial treatment option while considering mastectomy in advanced stages of the disease.^26,27^

The aim of our study was to assess the treatment approaches in patients with nonmetastatic breast cancer with regard to the administration of NACT and its effects on surgical practice in the Kurdistan region of Iraq and compare it with the current international guidelines. Because most patients with advanced breast cancer are still treated outside the MDT, our work intends to report the status of breast cancer treatment in the Kurdistan region of Iraq and advocates for an evidence-informed and MDT approach to deliver individualized, patient-centric shared treatment decision making.

## METHODS

### Patient Selection

This retrospective cohort study was based on data obtained from the medical records of female patients with breast cancer treated in oncology centers located in the Kurdistan region of Iraq. These centers include Nanakali Hospital for Blood Diseases and Cancer (Erbil), Rizgary Hospital (Erbil), Hiwa Hospital (Sulaymaniyah), and Azadi Teaching Hospital (Duhok).

This study included 1,000 patients with breast cancer diagnosed with primary, previously untreated, noninflammatory breast cancer (American Joint Committee on Cancer [AJCC] stage groups I, II, IIIA, and IIIC disease at diagnosis^5^) from the abovementioned oncology centers between January 2016 and December 2021.

Male patients with breast cancer with evidence of metastatic disease at presentation, locally recurrent disease, skin invasion, or T4 disease and those lacking clinical and/or histopathologic data were excluded. Patients with rare histologic subtypes (phyllodes tumor, breast sarcoma, and primary lymphoma of the breast) were also excluded.

Patients with conditions that would contraindicate radiation therapy, such as connective tissue disorders and a history of radiotherapy in the breast area, were excluded from this study.

The following clinicopathologic information was obtained and reviewed from the patient's medical records:

 1. Age at diagnosis, body surface area, and menopausal status.
2. Breast cancer molecular subtypes according to the 2013 St Gallen consensus.^28^
3. Tumor histology, grade, and tumor node metastasis staging were based on the AJCC Cancer Staging Manual, Eighth Edition.^5^
4. Local and systemic therapies, use of neoadjuvant systemic therapy, and chemotherapy with or without targeted therapy. Adjuvant systemic therapy, chemotherapy, targeted therapy, radiotherapy, and endocrine therapy.
5. Surgical approaches, that is, mastectomy or breast conservation surgery.
6. ALND or sentinel lymph node biopsy (SLNB).

### Confidentiality and Ethical Approval

All the identifying variables of the participants were removed. This study was approved by the Ethics Committee of the Ministry of Higher Education and Scientific Research, Hawler Medical University (approval No. 2/1466; May 17, 2021). Written informed consent was obtained from the participating hospitals to access patients' medical records. This was a low-risk investigation, and data were only collected as anonymized and presented as grouped; therefore, consent from a single patient was not requested on the basis of the local ethical policies on the medical research matter and conditions of the Ministry of Health approval.

### Statistical Analysis

The results were analyzed using the Statistical Package for Social Sciences (SPSS v26). Differences in patient age at breast cancer diagnosis, stage, lymph node status, and tumor characteristics were assessed using the chi-square test after categorical subdivision. A *P* value <.05 was regarded as statistically significant.

## RESULTS

### Patient and Tumor Characteristics

**FIG 1:**
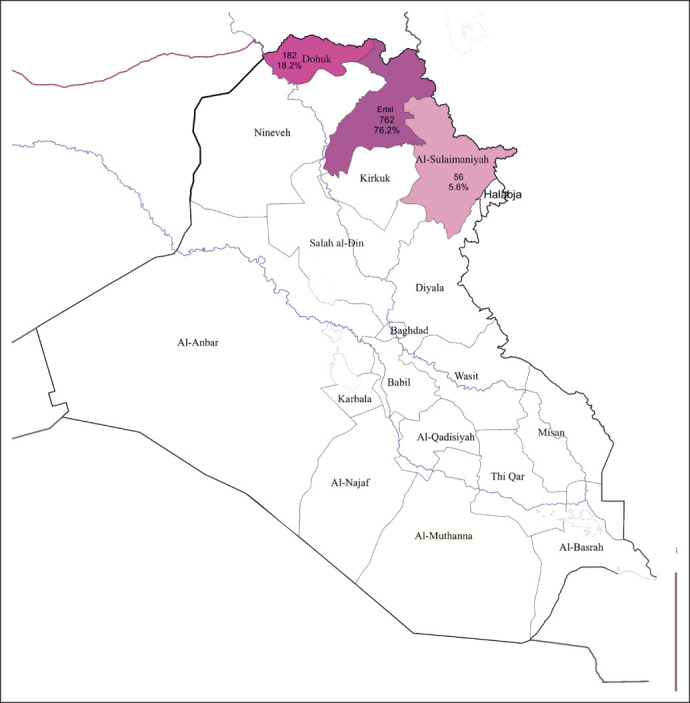
Iraq map showing the 18 Iraqi provinces including Erbil, Dohuk, and Sulaymaniyah, where the patients included in our study were diagnosed and treated from 2016 to 2021.

In the present study, after excluding missing data, 1,000 female patients from Erbil, Duhok, and Sulaymaniyah (Fig 1) who met the inclusion criteria in the specified period (2016-2021) were recruited.

**FIG 2:**
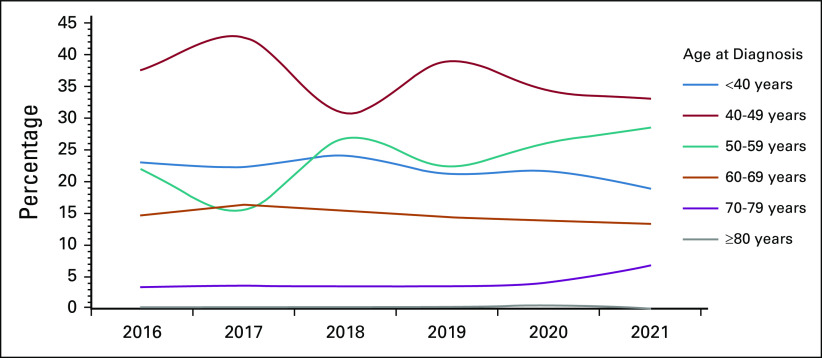
Age group distribution during 2016-2021.

The patient characteristics are summarized in Table 1. The median age at diagnosis was 47 years (range, 22-85 years). Patient age was categorized into groups <40 years, 40-49 years, 50-59 years, 60-69 years, 70-79 years, and ≥80 years. Most patients (35.8%) were age 40-49 years, one patient was older than 80 years, and approximately one fourth (22%) of the patients were younger than 40 years (Fig 2).

**TABLE 1:**
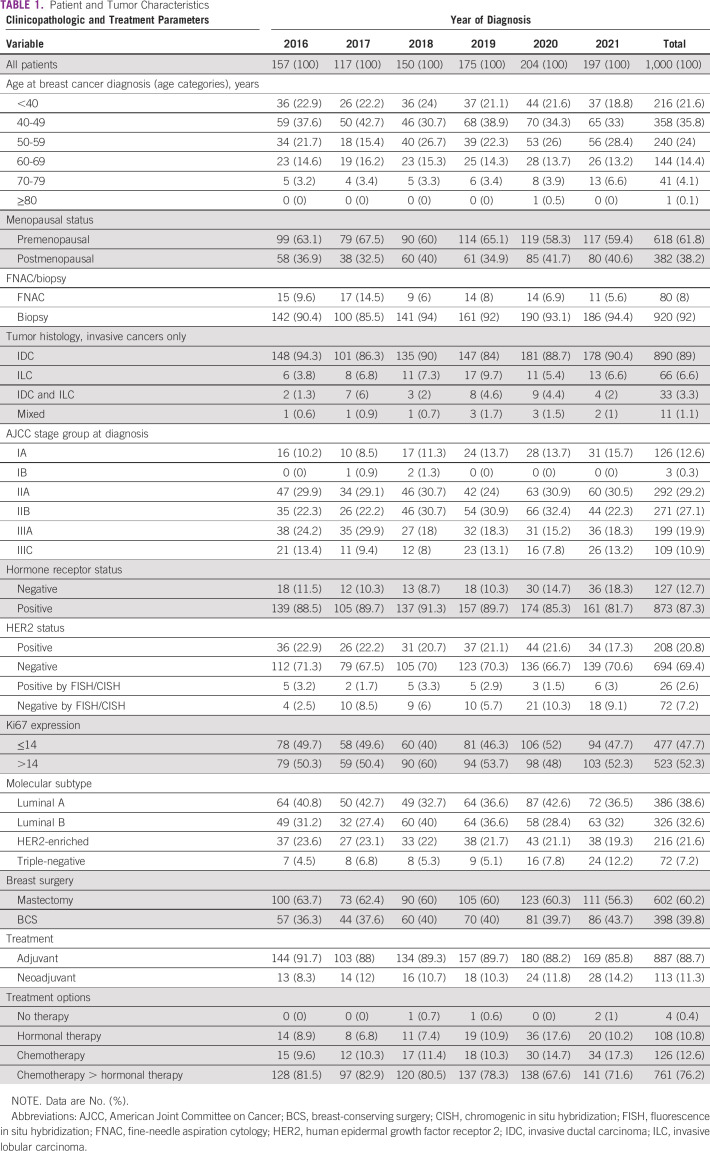
Patient and Tumor Characteristics

A majority of patients (61.9%) were premenopausal at the time of diagnosis, whereas the remaining (38.1%) were postmenopausal. For patients with BMI data available for analysis (652 patients in total), more than half of them (54.8%) had a mean BMI of 30.4 kg/m^2^.

Tru-cut biopsy was the most common method of pathologic diagnosis performed in 92% of the patients, which is of particular importance in the setting of NACT, whereas fine-needle aspiration cytology alone was used for cancer diagnosis in only 80 patients. Invasive ductal carcinoma was the most prominent histopathologic subtype, accounting for approximately 89% of all cases. The remaining histology represented a small percentage of invasive lobular carcinoma (6.6%) and mixed histology (4.4%).

**FIG 3:**
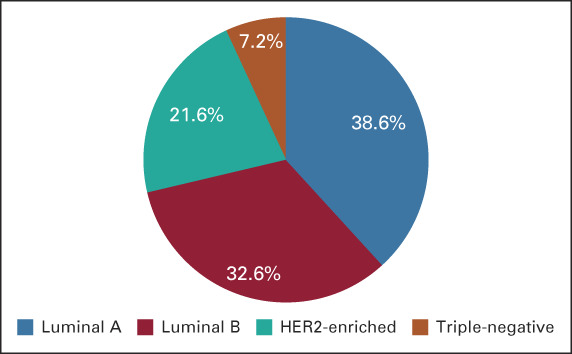
Immunohistochemical breast cancer subtype. HER2, human epidermal growth factor receptor 2.

HR (estrogen receptor [ER] and/or progesterone receptor [PR]) positivity was demonstrated in 87.3% of the patients (Table 1). Furthermore, HER2-overexpressing tumors (3+ by immunohistochemistry or amplified by in situ hybridization) were observed in 23.4% of patients. The molecular subtype categorization was as follows: luminal A (HR-positive, HER2-negative, and Ki-67 ≤14%) and luminal B (HR-positive, HER2-negative, and Ki-67 >14%) were the most common molecular subtypes reported in 38.6% and 32.6% of cases, respectively, whereas approximately 22% had HER2-enriched disease. Triple-negative breast cancer (TNBC; HR-negative, HER2-negative) was the least common cancer and was reported in only 7.2% of the study population (Fig 3). Ki67 was high (>14) in 52.3% and low (≤14) in 47.7% (Table 1).

**FIG 4:**
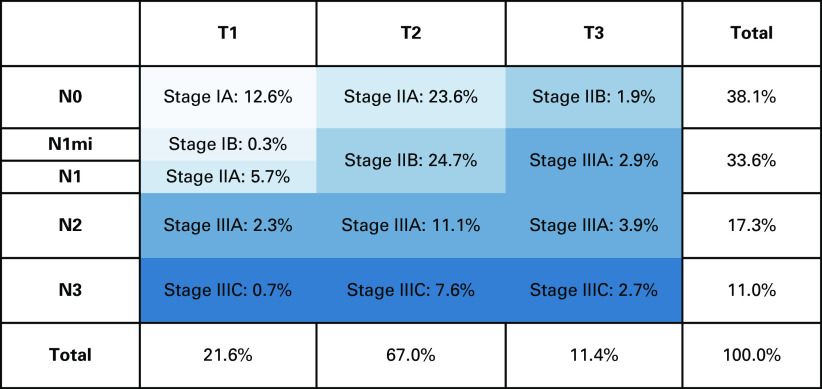
AJCC stage group at diagnosis. AJCC, American Joint Committee on Cancer; N, node; N1mi, micrometastasis of ≥2mm to 1 axillary node; T, tumor.

**FIG 5:**
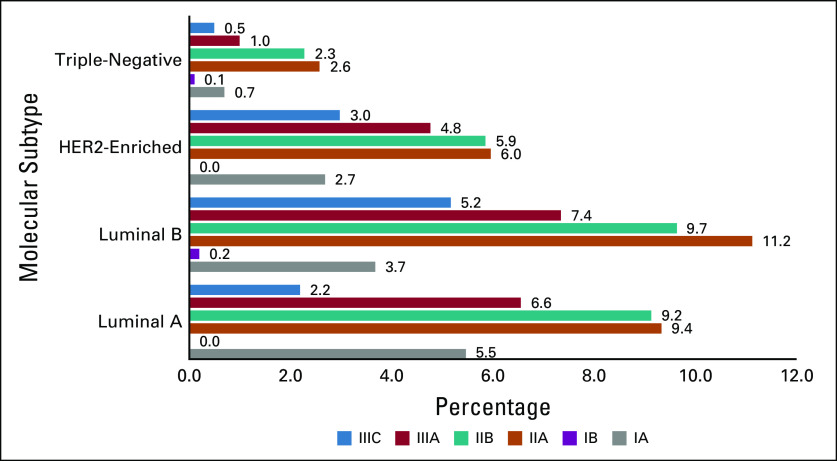
Distribution of molecular subtypes according to the stage of the disease. HER2, human epidermal growth factor receptor 2.

Among the total cases included in this study, approximately 13% had stage I disease (12.6% stage IA, 0.3% stage IB), 563 (56.3%) were diagnosed with stage II disease (29.2% stage IIA, 27.1% stage IIB), and 308 (30.8%) had stage III disease (19.9% stage IIIA, 10.9% stage IIIC; Fig 4). Both stages IIA and B were associated with higher ER- and PR-positive luminal A tumors (10.4% and 10.8%, respectively; Fig 5).

**FIG 6:**
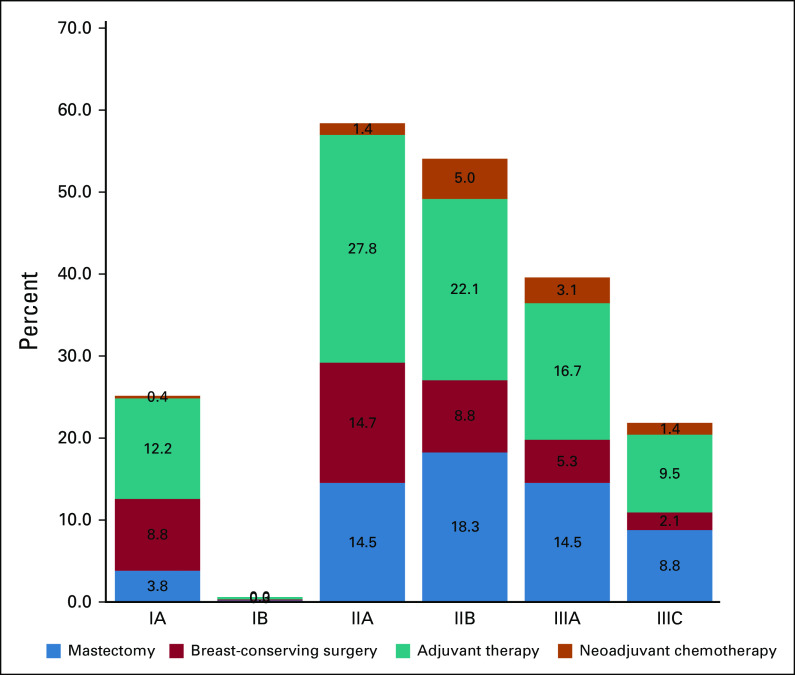
Local and systemic therapy patterns according to the stage of the disease.

Overall, 887 (88.7%) patients were administered adjuvant treatment and 113 (11.3%) patients received NACT (Fig 6). The use of adjuvant chemotherapy has decreased over time, particularly in women with HR-positive breast cancers. In total, 78.8% of patients received radiotherapy.

### Patient Characteristics and Definitive Surgery Information

In this study, female patients with breast cancer were classified into two groups: patients who underwent BCS followed by adjuvant radiotherapy and those who were elected for mastectomy. We evaluated both groups with regard to patient and disease characteristics, including age, BMI, tumor differentiation, pathologic type, molecular subtype, and site and location of the primary tumor within the breast; no significant differences were identified. Approximately 60% of patients underwent mastectomy, whereas the remaining (approximately 40%) patients underwent BCS (Table 2).

**TABLE 2:**
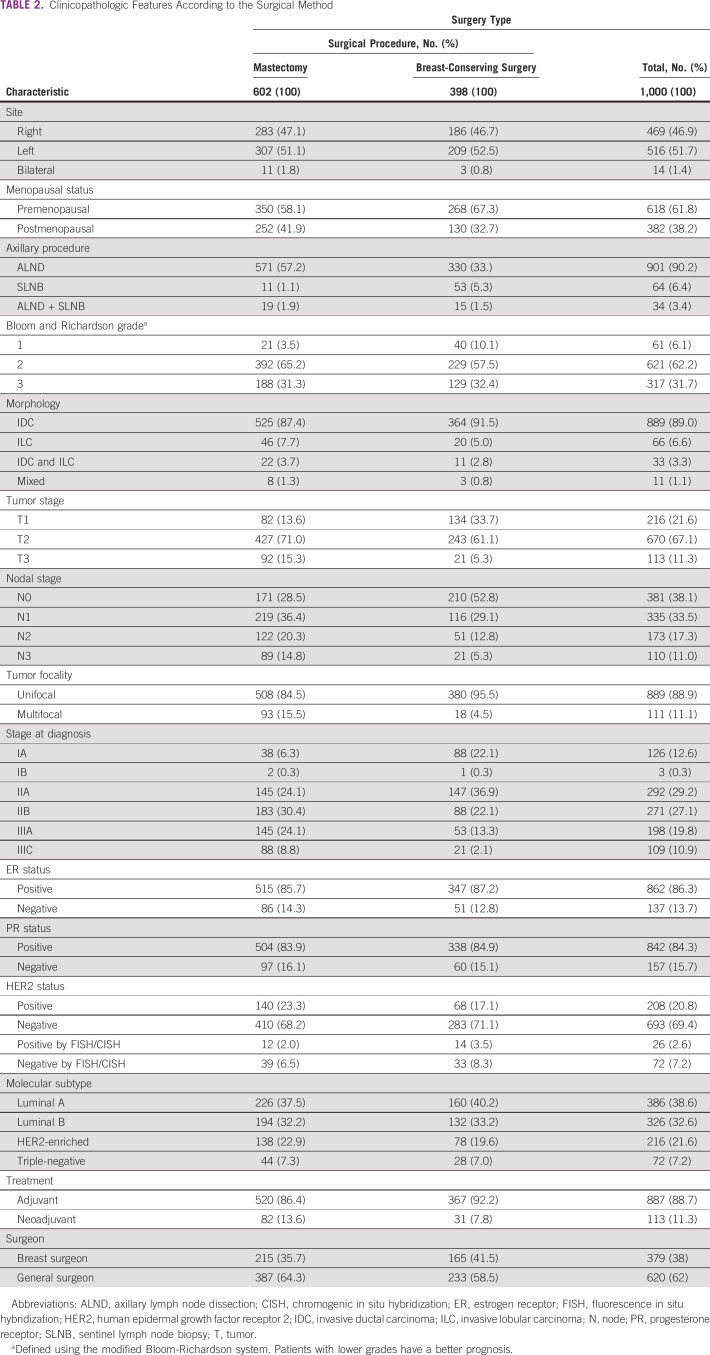
Clinicopathologic Features According to the Surgical Method

**FIG 7:**
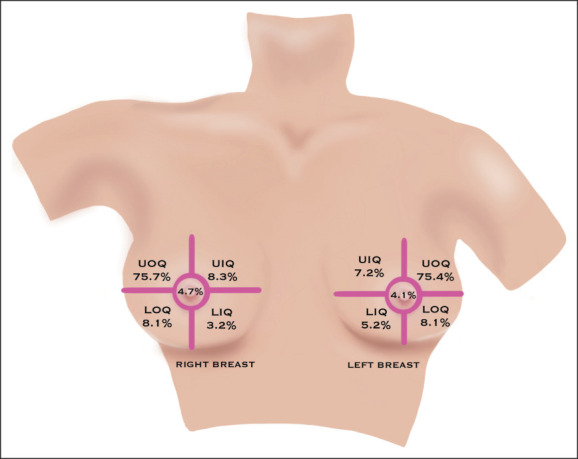
Site of primary tumor. LIQ, lower inner quadrant; LOQ, lower outer quadrant; UIQ, upper inner quadrant; UOQ, upper outer quadrant.

In this study, 51.6% of the women had tumors in the left breast. The primary tumor sites within the breast varied, with the highest frequency in the upper outer quadrant observed in 75.6% of the cases and the lowest frequency in the lower inner quadrant at 4.2% (Fig 7).

All the patients in this study underwent axillary lymph node staging. This was performed by either ALND or SLNB. In total, 94% of the patients underwent ALND upfront or after a positive SLNB and approximately 60% of the patients underwent mastectomy, compared with 34% of the patients who underwent BCS. Thirty-four patients with SLN-positive tumors underwent complete ALND. SLNB, without complete dissection, was performed in 64 patients.

### Characteristics of the Patient With Locally Advanced Breast Cancer

**FIG 8:**
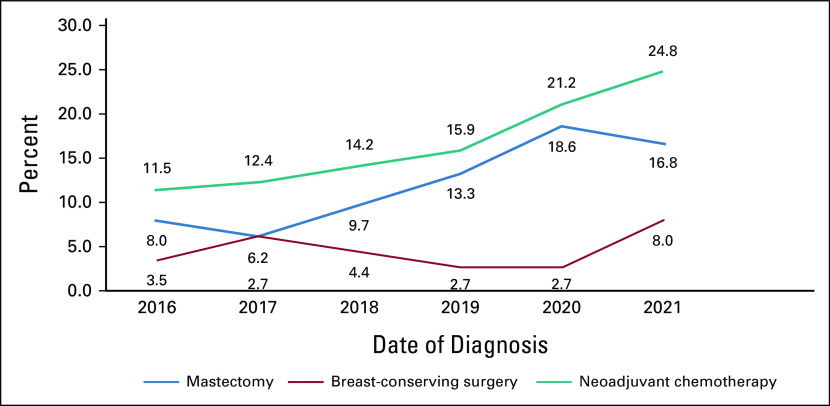
Correlation between neoadjuvant chemotherapy use and the breast surgery type during the period 2016-2021.

This study included 113 patients treated with NACT followed by breast surgery (Fig 8). T1 tumors accounted for 6%, T2 for 72%, and T3 for 22%. Regarding the clinical lymph node status, 13.2% were N0, 53.1% were N1, 21.2% were N2, and 12.3% were N3. Among the patients in the BCS group, a larger proportion (67.3%) were premenopausal, whereas 41.9% were postmenopausal in the mastectomy group.

Most patients with LABC were treated with primary surgery followed by adjuvant chemotherapy (83.5%), and the rest with NACT, among whom only 31 (27.4%) had undergone BCS compared with 82 (72.6%) who had undergone mastectomy (Fig 8).

Most patients who underwent BCS had operable LABC (cT2N1, 36.9%). The corresponding proportion of those who underwent mastectomy was 24%; 81 patients who received NACT underwent axillary dissection in the mastectomy group, and 29 patients who received NACT underwent axillary dissection in the BCS group.

A total of 68 patients received taxane- and anthracycline-based chemotherapy courses, whereas 45 patients with HER2-enriched cancer received chemotherapy plus target therapies, of whom eight patients received dual blockade anti-HER2 therapy; of them, six patients achieved pathologic complete response (pCR). The overall pCR rate in patients who received NACT was 29.2%. By contrast, 70.8% of the patients had a partial response on the basis of clinical criteria (tumor shrinkage, as reported by clinicians in the medical records).

In total, 62% of the breast surgeries were performed by general surgeons, and 38% by breast surgeons. Approximately 48% of patients who received NACT underwent BCS performed by a breast surgeon (Table 2).

## DISCUSSION

Breast cancer is the most common cancer among women worldwide and in Iraq.^29^ It is currently diagnosed and treated at an early stage in most developed countries.^30^ There are still great disparities in clinicopathologic profiles, stage at presentation, and treatment approaches for patients with breast cancer in developing countries.^31,32^

In the current study, LABC constituted approximately 58% of the new breast cancer cases in the Kurdistan region of Iraq, which is in agreement with other studies from developing countries.^33^ Previously published studies from Iraq conducted in 2016 and 2018 reported an incidence of advanced breast cancer stages at diagnosis of 47% and 67%, respectively.^34,35^ A recently published local study reported (46%) advanced-stage disease at presentation.^36^ This delayed presentation could be explained by various factors such as lack of education, poor awareness of cancer, lack of population screening programs, cultural barriers, and poor socioeconomic status.^37,38^

This study investigated the treatment approach in patients with LABC and found that 56% of women, most of whom (84%) underwent surgery first and 16% received neoadjuvant therapy. Most patients who received NACT underwent mastectomy, 82 (72.6%), rather than BCS, 31 (27.4%). Unfortunately, modified radical mastectomy remains the standard surgical approach in Iraq and other Arab countries.^34,39-41^

Trends in the use of BCS in our study have increased from 36.3% in 2016 to 43.7% in 2021. Many studies have reported that NACT reduces mastectomy rates. In the BrighTNess randomized clinical trial, more than 50% of the patients became BCS-eligible after NACT.^42^ In New York, 69% became BCS candidates between 2013 and 2019 at the Memorial Sloan Kettering Cancer Center.^43^ The BCS rate increased from 40.4% to 62.6% in a prospective study conducted in Seoul, Korea, between 2014 and 2015.^44^ In the Netherlands, BCS increased from 43% to 57% between 2011 and 2016.^45^

In this study, 113 patients with breast cancer were treated with NACT. The proportion of patients treated with NACT has increased over time, from 13 (8.3%) in 2016 to 28 (14.2%) in 2021, in line with international trends.^46^ Currently, systemic treatment is customized according to each breast cancer subtype and has moved toward NACT rather than adjuvant chemotherapy.^47^ The highest use of NACT was in HER2-enriched and TNBC in approximately 44% of the patients, whereas luminal types constituted 55.7% of the patients. The Ki-67 proliferation index was >14 in 73.4% of the patients.

Response to NACT was evaluated clinically, radiologically, and pathologically. Preoperative clinical and radiologic response evaluations were also performed. Although computed tomography and magnetic resonance imaging are best for evaluating the response to NACT owing to cost considerations, the response was measured clinically by physical examination and radiologically by ultrasonography. The pathologic response was assessed by measuring the tumor and lymph node sizes after NACT using the RECIST guidelines (version 1.1).^48^

The prognostic value of pCR after NACT depends on the molecular subtype of breast cancer^49^; TNBC and HER2-enriched patients have higher pCR rates than luminal breast cancer.^50^ Thirty-three (29.2%) patients achieved pCR, 9 (7.9%) had luminal breast cancer, 21 (18.6%) had HER2 overexpression tumors, and 3 (2.7%) had TNBC.^51,^^52^

Studies have shown an increase in the BCS rate after neoadjuvant treatment and because of the gradual expansion of treatment options to less toxic-targeted therapies.^14^ The conversion rate from mastectomy to BCS was more than 50% after NACT plus dual-target therapy in the Asian population.^53^ In our data, of 45 patients who received NACT plus target therapies, eight patients received dual blockade anti-HER2 therapy and six patients achieved pCR and then were treated with BCS.

Currently, the use of neoadjuvant therapy is associated with a lower need for extensive axillary lymph node treatment, especially for those who achieve axillary lymph node downstaging.^54^ Although all the patients included in our study underwent axillary surgery, the majority of them had ALND (94%) and only a minority had SLNB (6%), which further reflects the need for practice change in our region toward less aggressive axillary lymph node staging. The advantage of SLNB is that it reduces the number of lymph nodes removed, limits ALND surgical complications, and does not affect survival as the AMAROS trial confirmed that.^55^

Surgeon preference appears to play a significant role in the selection of patients for neoadjuvant therapy. In total, 62% of breast surgeries were performed by general surgeons and 38% were performed by breast surgeons. Among the total number of patients who received NACT and underwent BCS, 48% of them were treated by a breast surgeon.

In the current study, some factors such as the patient's choice and whether the surgeon discussed the possible surgical approach could not be assessed. In addition, none of these patients were discussed in a proper MDT before treatment. Furthermore, immunohistochemical analysis is not usually performed unless surgery is performed.

This study examines an important aspect of the treatment approach for 1,000 patients with breast cancer who underwent breast surgery, either mastectomy or BCS, and received either neoadjuvant or adjuvant treatment at oncology centers in the Kurdistan region of Iraq. In this study, an effort was made to collect detailed and accurate data, making our results applicable and useful for understanding breast cancer management in this region. The most important limitation of this study was the retrospective study design with limited data on potentially important factors, such as performance status, detailed comorbidities, patients' wishes, and prior discussion of possible surgical approaches by the surgeon.

In conclusion, our study concluded that temporal trends in the timing of systemic chemotherapy delivery have changed in recent years, with the use of NACT along with BCS in LABC increasing in the Kurdistan region, in line with the international guidelines. However, in our study, mastectomy was more common than BCS. This emphasizes the urgent need to establish functional MDT to determine the best treatment approach for each breast cancer case. In addition, increasing awareness of breast cancer, effective breast cancer screening programs, and early detection are important factors in aiding to move toward BCS.
